# Reference-free structural variant detection in microbiomes via long-read co-assembly graphs

**DOI:** 10.1093/bioinformatics/btae224

**Published:** 2024-06-28

**Authors:** Kristen D Curry, Feiqiao Brian Yu, Summer E Vance, Santiago Segarra, Devaki Bhaya, Rayan Chikhi, Eduardo P C Rocha, Todd J Treangen

**Affiliations:** Department of Computer Science, Rice University, 6100 Main St., Houston, TX 77005, United States; Department of Genomes and Genetics, Microbial Evolutionary Genomics, Institut Pasteur, Université Paris Cité, CNRS, UMR3525, Paris 75015, France; Arc Institute, Palo Alto, CA 94304, United States; Department of Environmental Science, Policy, and Management, University of California, Berkeley, CA 94720, United States; Department of Electrical and Computer Engineering, Rice University, Houston, TX 77005, United States; Carnegie Institution for Science, Department of Plant Biology, Stanford, CA 94305, United States; Department of Computational Biology, Institut Pasteur, Université Paris Cité, Paris 75015, France; Department of Genomes and Genetics, Microbial Evolutionary Genomics, Institut Pasteur, Université Paris Cité, CNRS, UMR3525, Paris 75015, France; Department of Computer Science, Rice University, 6100 Main St., Houston, TX 77005, United States

## Abstract

**Motivation**: The study of bacterial genome dynamics is vital for understanding the mechanisms underlying microbial adaptation, growth, and their impact on host phenotype. Structural variants (SVs), genomic alterations of 50 base pairs or more, play a pivotal role in driving evolutionary processes and maintaining genomic heterogeneity within bacterial populations. While SV detection in isolate genomes is relatively straightforward, metagenomes present broader challenges due to the absence of clear reference genomes and the presence of mixed strains. In response, our proposed method rhea, forgoes reference genomes and metagenome-assembled genomes (MAGs) by encompassing all metagenomic samples in a series (time or other metric) into a single co-assembly graph. The log fold change in graph coverage between successive samples is then calculated to call SVs that are thriving or declining.

**Results**: We show rhea to outperform existing methods for SV and horizontal gene transfer (HGT) detection in two simulated mock metagenomes, particularly as the simulated reads diverge from reference genomes and an increase in strain diversity is incorporated. We additionally demonstrate use cases for rhea on series metagenomic data of environmental and fermented food microbiomes to detect specific sequence alterations between successive time and temperature samples, suggesting host advantage. Our approach leverages previous work in assembly graph structural and coverage patterns to provide versatility in studying SVs across diverse and poorly characterized microbial communities for more comprehensive insights into microbial gene flux.

**Availability and implementation**: rhea is open source and available at: https://github.com/treangenlab/rhea.

## 1 Introduction

Structural variants (SVs), loosely defined as genomic alterations that are 50 base pairs (bps) or longer (Mahmoud *et al.* 2019), play an important role in driving both evolutionary adaptation and heterogeneity in bacterial genomes (Rocha 2018). Bacterial genome dynamics not only influence the ability for the bacteria to grow and adapt to changing environments (Rocha 2004) but can also impact the function of the microbial community as a whole and the phenotype of the host (Durrant and Bhatt 2019). In isolate genomics, the goal of SV detection is relatively straightforward: detect long genomic differences between a sequence and reference genome that can be classified as an insertion, deletion, inversion, duplication, translocation, or any combination of the prior (West *et al.* 2022). However, in metagenomics, when reference genomes may not be well-defined and a mixed population of similar strains may exist in the community, detection of SVs becomes more complex (West *et al.* 2022).

SV detection methods can be broadly categorized into three groups: mapping-driven, assembly-driven, and pattern-driven. In mapping-driven approaches, reads are directly aligned to established reference genomes or pangenome of sequences, then, mapping patterns signifying inconsistent coverage identify SVs. In assembly-driven approaches, reads are first assembled into longer sequences (contigs), then aligned to another contig or reference to detect long scale differences. In pattern-driven approaches, SV patterns are pre-defined then searched for in sequencing reads. Zeevi *et al.* developed a mapping-driven SV detection approach for metagenomic short reads to survey SVs associated with host disease risk factors in the human gut microbiome (Zeevi *et al.* 2019). The authors built a comprehensive database specifically for known microbes in the human gut microbiome and developed an “iterative coverage-based read assignment” (ICRA) algorithm to repeatedly adjust read assignments and establish alignments. Their SGV-Finder algorithm then scans the coverage of each reference genome for presence of regions with unexpectedly low (deletions) or high (duplications) coverage. While this method has been effective as a comprehensive search for SVs in the human gut microbiome correlating to expressed phenotypes (Liu *et al.* 2023), relying on a confident database of reference genomes is challenging for communities that have not been extensively characterized. This pipeline is additionally restricted to only deletions and duplications relative to reference genomes in the supplied database.

To expand upon the types of SVs detected and leverage the advantages of long read technologies, MetaSVs, an assembly-driven approach, was designed (Li *et al.* 2023). In this pipeline, long and short reads combined help to confidently create and classify metagenome-assembled genomes (MAGs). Each MAG is then evaluated independently through whole-genome alignment to a reference MAG or genome with the SV detection tool MUM & Co (O’Donnell and Fischer 2020). Chen *et al.* utilized MetaSVs to expand characterized SVs in the human gut (notably insertions and inversions) and demonstrates the value in incorporating long reads for SV detection (Chen *et al.* 2022). However, this assembly-driven method is still highly dependent on a reference database, as it is the taxonomic reference-driven classifications that determine which MAGs are compared to which references. Additionally, unique MAGs are often not created for subtle SV differences (Kerkvliet *et al.* 2024), especially in communities containing similar strains (Ghurye *et al.* 2016).

MetaCHIP is another MAG-based approach for the slightly different goal of detecting recent horizontal gene transfer (HGT) events (Song *et al.* 2019). In an HGT event, genetic material is exchanged between organisms (Ochman *et al.* 2000), resulting in an insertion SV for the recipient. MetaCHIP effectively evaluates each MAG in the community for a gene sequence that has more BLASTN (Altschul *et al.* 1990) hits to genes in a different MAG than its own. This algorithm, however, can only detect inserted genes that are highly similar to another MAG, which resulted in simulation results declining at 25% mutation rate between donor and recipient.

To entirely avoid reference genomes and MAGs, two pattern-driven methods have been developed. PhaseFinder (Jiang *et al.* 2019) was created for detection of inversions in bacterial genomes from genomic or metagenomic data, by detecting regions flanked by inverted repeats where sequencing reads support both orientations. DIVE (Abante *et al.* 2023) was developed to identify sequences surrounding genetic diversification such as transposable elements, within mobile genetic element (MGE) variability hotspots, or CRISPR repeats, by detecting repeated k-mers with diverse flanking sequences to define MGE bounding sequences and transposon arms. Both these methods show how detection of specific patterns directly from reads can be used to eliminate reference genomes and MAGs.

Rhea takes a different approach to detect SV patterns within a microbial community. It constructs a co-assembly graph from all metagenomes in a series that are expected to have similar communities (i.e. longitudinal time series or cross-sectional studies where a significant portion of the strains are shared across samples) (Quince *et al.* 2021). Regions of the graph indicative of SVs are then highlighted, as previously explored for characterization of genome variants (Iqbal *et al.* 2012, Nijkamp *et al.* 2013, Narzisi *et al.* 2018, Ghurye *et al.* 2019). The log fold change in graph coverage between consecutive steps in the series is then used to reduce false SV calls made from assembly error, account for shifting levels of microbe relative abundance, and ultimately permit SV detection in understudied and complex microbiomes.

## 2 Materials and methods 

### 2.1 Rhea method

**Figure 1.**
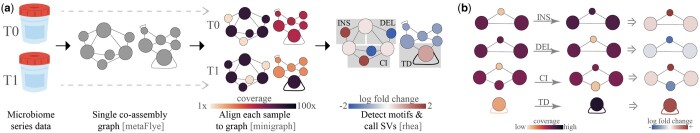
(a) Rhea takes a series of long-read metagenomic reads as input. Then, a co-assembly graph of all reads is created with metaFlye. Reads from each sample are then separately aligned to the co-assembly graph with minigraph. Rhea evaluates log fold change in coverage between series steps for SV-specific patterns in the assembly graph to detect SVs between steps. (b) Assembly graph patterns detected in rhea, which indicate insertions (INS), deletions (DEL), complex indels (CI), and tandem duplicates (TD). INS and DEL are detected by observing a triangle where one node has a significantly higher (INS) or lower (DEL) log fold change. CIs are noted by a square with one or two outliers; in the case of two outliers, the two outliers must be of opposing sides of the median and not have an edge between them. TDs are detected by a log fold change of a self-loop edge coverage greater than 1.

Rhea takes as input a series of long-read metagenomic sequences, expected to be taken from the same source at different time points or some other step-wise metadata separation. A single metagenome assembly graph is constructed by combining all provided samples, then each sample is separately aligned back to the graph. Change in graph coverage between consecutive pairs of samples and the graph structure are used to call SVs (Fig. 1). If desired, quality filtering or read removal should be completed prior to rhea’s graph construction.

#### 2.1.1 SV definitions

Four types of SVs are detected in rhea: insertions, deletions, tandem duplications (West *et al.* 2022), and complex indels (Roerink *et al.* 2014, Ye *et al.* 2016). An insertion here is a sequence that has been integrated in increasing abundance between successive steps in the sequential series. A deletion is the opposite, a subsequence whose abundance is declining. A tandem duplication is a gene sequence that has been repeated, directly one after another, in increasing presence. A complex indel as a sequence that has drastically changed between successive steps, showing the signature of a deletion and insertion at the same location. In this pipeline, SV detection equates to an increase in abundance of the SV, rather than simply a novel appearance, therefore, suggesting an advantage for the host microbe or community.

#### 2.1.2 Graph construction and coverage calculations

A single co-assembly repeat graph for the series with *N* samples is constructed by combining all reads from all samples into one metaFlye run (Kolmogorov *et al.* 2020), with—keep-haplotypes parameter set to true to maintain strain variations. After the graph is constructed, each sample is separately aligned back to the graph with minigraph (Li *et al.* 2020), where the majority of the reads are expected to align to the graph since all reads were included in graph construction. An undirected graph is then built mimicking the structure of the metaFlye assembly graph where a single node is drawn for each complementary pair, as seen in the assembly graph visualization software Bandage “single” option (Wick *et al.* 2015). This graph is defined as G=(V,E) with a set of *k* nodes $V=\{v_{1},v_{2}..,v_{k}\}$ and a set of edges *E*. Each edge $\left(e_{i,j}\right)$ is then given a weight equal to the number of edges that appear between nodes *i* and *j* in the metaFlye assembly graph, given there exist at least one edge between *i* and *j* in the assembly graph. Each edge $\left(e_{i,j}\right)$ thus denotes the existence of overlap reads that expand directly from $v_{i}$ to $v_{j}$ (or from $v_{j}$ to $v_{i}$) without gaps, in either direction (forward or reverse) for the sequences in *i* and *j*. Minigraph alignments are then used to calculate node and edge coverage for each step in the series. Node coverage is calculated as the average coverage per base pair within the node, calculated by summing the coverage for each base pair divided by the total number of base pairs in the node. To account for error, all nodes with coverage less than 1, are set to a coverage of 1. Node coverage is then normalized for the entire series, by first calculating the median total base pairs *m* across samples in the series, then establishing a multiplier for each sample n=0..N as $bp_{n}/m$, where $bp_{n}$ is the number of base pairs in sample *n*. This multiplier for each step is applied to all node coverages for each n=0..N. Edge coverage for each edge $e_{i,j}$ at each step *n* in the series is counted as the number of occurrence a read path covers directly from *i* to *j* or *j* to *i* in the read-graph alignment for step *n*. Each node in our undirected assembly graph then holds a vector of log fold change in coverage between successive steps in the series, calculated for each node *i* as $\log(vc_{i,t_{n}}/vc_{i,t_{n-1}})$, where $vc_{i,t_{n}}$ is the coverage of node *i* at step *n* in the series for all steps n=1…N. A log fold change vector is also assigned to each edge (i,j), defined as $\log(ec_{(i,j),t_{n}}/ec_{(i,j),t_{n-1}})$, where $ec_{i,t_{n}}$ is the coverage of edge $e_{i,j}$ at step *n* in the series for all steps n=1..N. The log fold change vectors are then used in the next step to detect SVs and account for assembly error and changes in genome relative abundance between successive samples.

#### 2.1.3 Detected SV graph patterns

Rhea utilizes the graph structure, edge weights, and the log fold change coverage vectors to call SVs between each pair of consecutive samples in the series. All triangles and squares, cycles of lengths 3 and 4, respectively, are detected in the co-assembly graph using NetworkX simple_cycles(length_bound=4) function (Hagberg *et al.* 2008, Gupta and Suzumura 2021). This function yields complexity $O((c\;+\;n)(k\;-\;1)d^{4})$, where *n*, *e*, and *c*, are the number of nodes, edges, and simple circuits, respectively, and *d* is the average degree of nodes. For insertions and deletions, each triangle is searched for the pattern of two similar log fold change values and one that is significantly different for each step. This is completed by: calculating the median and standard deviation between the three log fold changes, then, labeling any node with a value that is more than one standard deviation away from the median as an outlier. If the triangle contains exactly one outlier, then an insertion or deletion is called, depending on if the outlier value is lower (deletion) or higher (insertion) than the median. Median is used here rather than mean to provide robustness against extreme outliers. For example, in the case of an extreme outlier due to a deletion from a thriving member in the community, the mean would be skewed and thus could call all three nodes an outlier; whereas the median would take the value of one of the non-deletion nodes, and thus, given the two non-deleted nodes carry a similar value, only the deletion would be an outlier. A similar process is conducted to search for complex indels. Here, each square in the graph is searched for outliers. If the square either has a single outlier or two outliers that do not have an edge between them (opposites in the square) and one is greater than the median while the other is smaller, a complex indel is called. A tandem duplicate can be called under two different scenarios. The first, a self-duplicate, shown by an edge log fold change of any self-loop edge greater than 1 for any successive steps in the series. The second is the situation where the duplicate produces a second node containing a nearly duplicate sequence and loops between two nodes. This is detected by searching all edges with weight w ≥ 2 for a log fold change edge weight greater than 1. If these criteria are met, the node with the greater log fold change coverage between the two is then called a tandem duplication, if it has not been called for another SV at the specified step.

### 2.2 Experiments

#### 2.2.1 Simulated HGT events

**Figure 2.**
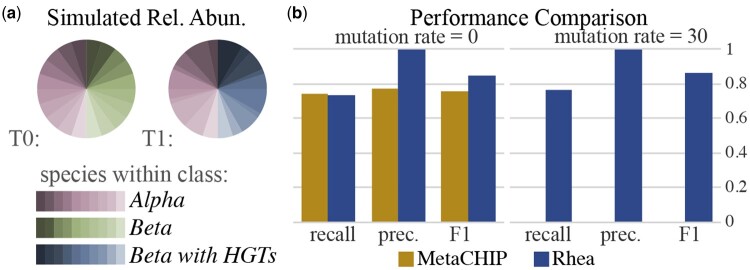
(a) Simulated relative abundances for time points *T*0 and *T*1. *T*0 is a simulation of the 20 reference genomes in equal abundance; *T*1 is simulated from the 10 original *Alphaproteobacteria* species and the 10 mutated *Betaproteobacteria* species in varying abundances (b) Precision, recall, and F1-score for MetaCHIP (Song *et al*. 2019) and rhea detected insertions for the mock community with mutation rates 0 and 30. Time point *T*1 is used for MetaCHIP results; change from *T*0 to *T*1 is used for rhea.

Rhea was compared to the metagenome HGT detection tool MetaCHIP by simulating long reads from the simulated HGT events completed in the HgtSIM manuscript (Song *et al.* 2017). For this community, 10 strains within class Alphaproteobacteria and 10 strains within class Betaproteobacteria were selected. 1 gene was selected from each Alphaproteobacteria, mutated with rate m, and inserted randomly into each Betaproteobacteria. This resulted in a total of 100 HGT events for the community (Fig. 2a). Three long read metagenomic datasets of 500,000 reads were simulated from these reference genomes with NanoSim (Yang *et al.* 2017) v3.1.0 with default parameters: a pre-transfer community (*T0*) of the 20 reference genomes in equal abundance, and two separate post-transfer communities with mutation rate m=0 and m=30 ($T1_{m0}$, $T1_{m30}$), which include the 10 original Alphaproteobacteria and the 10 HGT-inserted *Betaproteobacteria* references in varying abundances (Fig. 2a). These varying abundances were established by randomly selecting a relative quantity between 1 and 5 for each of the species as input into the NanoSim abundance text file. MetaCHIP v.1.10.12 was run with GTDB-Tk (Chaumeil *et al.* 2022) v2.2.6 with taxonomy release 207 and -r set to class (c). Rhea v1.0 was run with default parameters, metaFlye v2.9.3, and minigraph v0.20. Simulated HGT insertions were mapped against reported HGT sequences for both methods using minimap2 (Li 2016) v2.24 with default parameters; each HGT insertion sequence was marked as detected if the sequence had a hit to a reported HGT insertion.

#### 2.2.2 Simulated SVs

**Figure 3.**
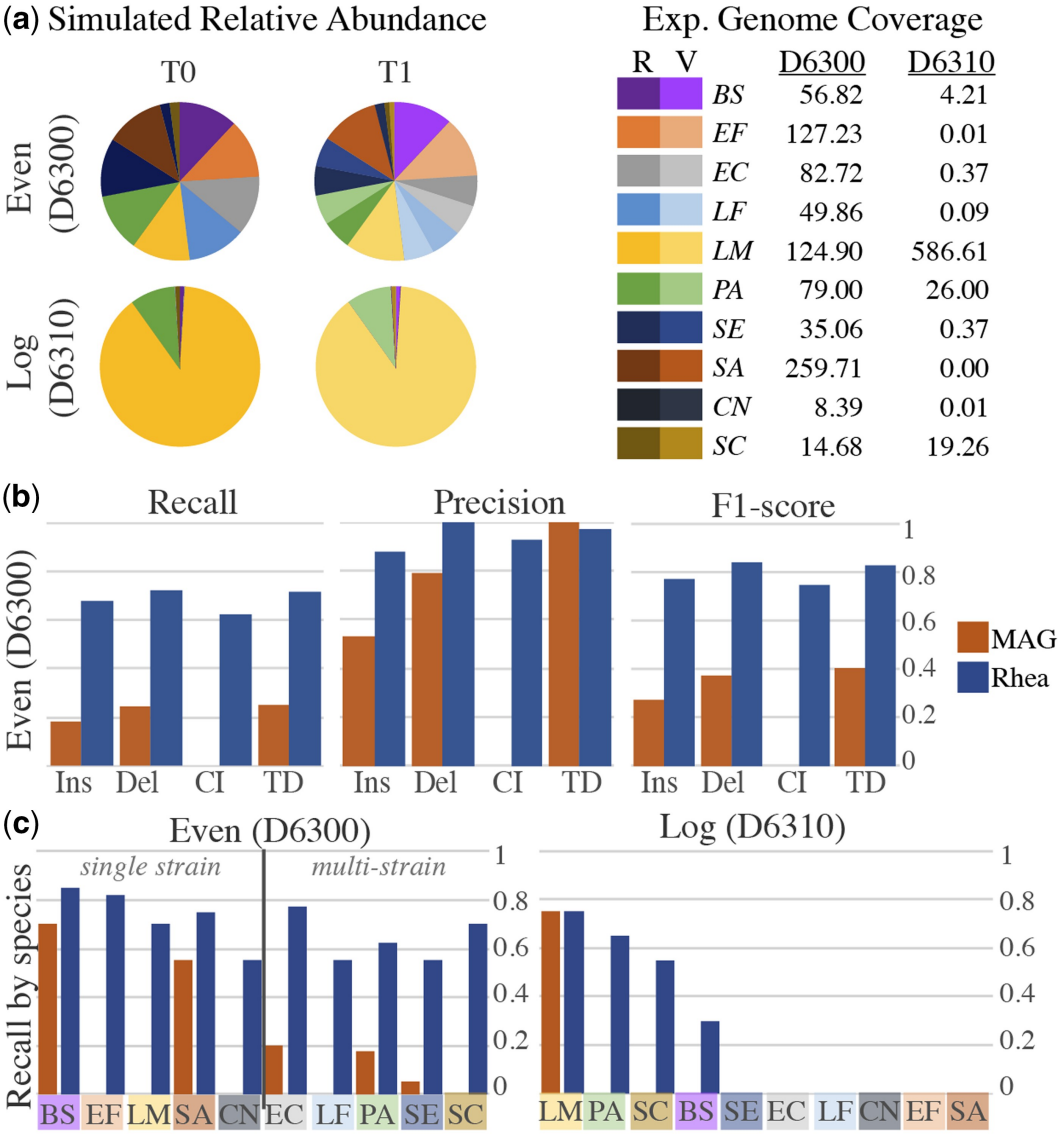
(a) Relative abundance of long reads for two simulated time points (*T*0, *T*1) for each of our ZymoBIOMICS communities, one with even distribution (D6300) and the other with log distribution (D6310). Each of the 10 microbes were randomly given 20 indels, 10 tandem duplications, and 10 long complex indels to create a variant strain (Jeffares *et al*. 2017). *T0* contains only the original references (R); *T1* introduces the variants (V), where, in our even distribution, half the species have variants in equal abundance to their original reference *[Escherichia coli (EC), Lactobacillus fermentum (LF), Pseudomonas aeruginosa (PA), Salmonella enterica (SE), Cryptococcus neoformans (CN)]*, and half the species are dominated by their variants *[Bacillus subtilis (BS), Enterococcus faecalis (EF), Listeria monocytogenes (LM), Staphylococcus aureus (SA), Saccharomyces cerevisiae (SC)]*. In our log distribution, only the variant strains are present. Expected genome coverage here is the expected read coverage across the entire length of the genome (total number of simulated from the reference * average read length/length of the reference genome). (b) Recall, precision, and *F*1-score for each of the SV types (Ins: insertion, Del: deletion, CI: complex indel, TD: tandem duplication) for both workflows in our even distribution. For the MAG workflow, MAGs were curated for *T*0 and *T*1 separately. Then, Mum & Co called SVs between *T*0 and *T*1 MAGs of matching taxonomic classification. (c) Combined recall for all SV types, separated by each species. For our even distribution, species are separated into two groups, signifying the presence of only the variant strain (single strain) or both the original and variant references (multistrain) at time point *T*1. For our log distribution, species are ordered by decreasing coverage.

To evaluate the accuracy of rhea for detection of SV types insertion, deletion, complex indel, and tandem duplication in comparison with a MAG-based workflow, two experiments mimicking the 10 microbes in ZymoBIOMICS Microbial Community Standard D6300 (even distribution) and D6310 (log distribution) were completed. SURVIVOR (Jeffares *et al.* 2017) v1.0.7 was used to randomly create 20 indels (insertions or deletions) and 10 tandem duplicates of length 500–2000 base pairs, with homozygous_ratio = 0.5 and Number_haploid = 1 in the parameters file, for each of the 10 reference genomes independently. Then, a custom script introduced 10 random complex indels of the same length range into each of the variant strains. The custom script randomly selected a location along the genome, then, performed a deletion and a random insertion, each within the prescribed length range. For our even distribution, two long read metagenomic datasets of roughly 500,000 reads were simulated from these reference genomes with NanoSim: a pre-transfer community (*T*0) of the original references in their provided relative abundances and a post-transfer community (*T*1), which includes only the variant strain for half of the species and equal abundance of variant and original strains for the other half (Fig. 3a). This was completed again for our log distribution, where only the original references were present in *T*0 and only the strains containing the added SVs in *T*1. The expected genome coverage for each species *s* was calculated for each distribution as $\frac{n_{s}*avg_{r}}{\mathrm{len}(s)}$, where $n_{s}$ is the number of read for species *s*, $avg_{r}$ is the average read length for the entire simulation, and len(s) is the length of the reference genome for species *s*. For our MAG workflow, reads were assembled with metaFlye (Kolmogorov *et al.* 2020) with—keep-haplotypes set to true, contigs were binned with MetaBat (Kang *et al.* 2019) v2.15 with default parameters, and bins were classified with GTDB-Tk. Bins with the same classification in both simulated samples were analyzed for SVs with MUM & Co (O’Donnell and Fischer 2020) v3.8 with the known reference genome length for parameter -g. Simulated SV sequences were mapped against reported SV sequences for both methods using minimap2. Each simulated SV was marked as detected if the sequence had a hit to a reported SV sequence with the correct SV type. Since MUM & Co does not call complex indels, we considered these correct if both the deletion sequence and the insertion sequence were returned.

#### 2.2.3 Cheese rind ripening

To evaluate rhea on a real microbiome, PacBio HiFi metagenomic reads from cheese rinds throughout ripening were taken from a previous study (Saak *et al.* 2023). One rhea run for “Cheese C” was completed with the 5 corresponding samples in temporal order and parameter—type set to pacbio-hifi. The selected assembly graph connected component was classified with GTDB-Tk (Chaumeil *et al.* 2022) “classify-wf” with default parameters, and is referred to as the *Halomonas* subgraph per this taxonomic classification. Mobile genetic element (MGE) contigs and putative hosts were established in the original publication utilizing Hi-C sequencing technology, overlap read coverage, and the viralAssociatePipeline (Bickhart *et al.* 2019). To determine which of these contigs showed signatures in our *Halomonas* subgraph, BLAST (Altschul *et al.* 1990) was run for all MGE contigs with a putative host, against the extracted *Halomonas* subgraph sequences as reference with default parameters. MGE contigs were considered to have their signatures present in the graph if a hit with query coverage >5% was reported. One subsection of the *Halomonas* subgraph was selected for further investigate as it showed a change in dominating graph path over time. Nodes within this path were characterized with SeqScreen-Nano (Balaji *et al.* 2023) v4.1 with default parameters and provided SeqScreen databases v21.4.

#### 2.2.4 Hot spring microbial mat sequencing

Microbial mat plugs were extracted from Mushroom Spring, Yellowstone National Park, USA on July 30, 2009 across a series of temperatures: 50°C, 55°C, 60°C, 65°C. DNA was quantified using the Qubit 3.0 Fluorometric Quantitation dsDNA High Sensitivity kit (ThermoFisher Scientific, Waltham, MA, USA) and stored for future use at −80°C. DNA extractions were analyzed using the Genomic DNA ScreenTape Analysis kit on the 4150 TapeStation System (both from Agilent, Santa Clara, CA, USA). Size selection using AMPure XP beads (Beckman Coulter, San Jose, CA, USA) increased DNA fragment length from a mean of 2 kb up to 6 kb with high recovery of DNA. Size selected DNA was prepped for sequencing using the Oxford Nanopore Technologies (ONT) 1D Genomic DNA by Ligation library preparation kit (SQK-LSK109, Oxford Nanopore Technologies, Oxford, UK). Libraries were then sequenced using the ONT MinION sequencer using one FLO-MIN106D R9 Version Rev D flow cell per temperature sample. Sequencing was run on a MacBook Pro (model A1502, Apple) using ONT’s MinKNOW software. Automatic basecalling through this software was turned off. Sequencing runs lasted between 24 and 44 hours. Basecalling was completed using the ONT software Guppy (https://github.com/nanoporetech/pyguppyclient.git) with default parameters.

#### 2.2.5 Hot spring microbial mat analysis

Rhea was run on Oxford Nanopore Technologies (ONT) reads from a hot spring microbial mat for 4 unique temperatures (see above) to asses an environmental microbiome with a high-level of complex microbial interactions (Bhaya *et al.* 2007, Nelson *et al.* 2011). Basecalled sequences were listed in order of increasing temperature with the—collapse parameter set to true. MAGs were also curated for reads from the 60°C sample by metaFlye assembly with—keep-haplotypes set to true and contigs binned with MetaBat 2 (Kang *et al.* 2019). Each read was then aligned back to the set of MAGs with minimap2 with default parameters. Reads with an alignment to a MAG contig of >80% of length were considered to be included in MAGs, mimicking the pipeline of a previous manuscript (Benoit *et al.* 2024). Kraken 2 (Wood *et al.* 2019) v2.1.1 was additionally run with the Kraken 2 default parameters and RefSeq indexes released on May 17, 2021 for all raw reads in this sample (constructed from 107 455 genomes).

## 3 Results

### 3.1 Simulated HGT insertions

Two simulation experiments were conducted with a community of strains within *Alphaproteobacteria* and *Betaproteobacteria* classes to evaluate HGT detection accuracy: one with mutation rates m=0 and the other with m=30. For the HGT insertions with m=0, rhea delivered comparable recall to MetaCHIP (0.73 to 0.74) and improved precision (1.0 to 0.77) (Fig. 2b). The only non-insertion SV that rhea called was a single complex indel, which was due to two insertions sequences in close genomic proximity. Given the two inserted sequences were still detected as sequences of increasing abundance, this was still considered this an accurate call. Although results for MetaCHIP and rhea for m=0 were relatively similar, a large discrepancy was observed for mutation rate m=30. Here, the accuracy for rhea stays consistent to that of no mutations (0.76 recall and 1.0 precision), yet MetaCHIP is not able to detect any of the HGT insertions. This caveat is also highlighted in the MetaCHIP manuscript; the inserted sequence is required to be present in another MAG (putative donor) in the community for MetaCHIP to be able to detect the HGT insertion. Additionally, MetaCHIP returned a total of 13 false positive insertions, while rhea did not report any false positives.

### 3.2 Simulated structural variants

Two simulated experiments were conducted to evaluate rhea in comparison to a MAG-based workflow for a variety of SVs. Each experiment contained two mock time points (*T*0 and *T*1), where *T*0 contains only the references in the ZymoBIOMICS Microbial Community Standard. For our even abundance distribution, *T*1 contains a mix of original references and simulated variant strains, while *T*1 contained only the simulated variant strains. For the even distribution, rhea greatly outperformed the MAG workflow in terms of recall (Fig. 3b). While rhea detected 71, 68, 63, and 72 of the simulated insertions, deletions, complex indels, and tandem duplications, respectively, the MAG workflow only identified 19, 23, 0, and 25, respectively. This discrepancy was largely due to the inability to curate independent MAGs for low abundant species and SV distinctions.

MAGs were classified for 5 of the 10 species at both *T*0 and *T*1, limiting the MAG-based workflow to only attempt to call SVs for these species. Of the five species, two (*B. subtilis, S. aureus*) were from species where the SV-containing strain dominated in sample *T*1, while three (*E. coli, P. aeruginosa, S. enterica*) contained both the original and the SV-containing strains in *T1*. Accuracy results between the rhea and MAG pipeline proved comparable for insertions, deletions, and tandem duplicates when only the SV-strain was present in post-transfer sample *T1*. However, when both the original and SV-strains were present, only one MAG was curated for the species, leaving many of the SV graph nodes unbinned and thus impossible to detect (Fig. 3c). To get a sense of the coverage needed for SV detection in each workflow, recall for each species was reported for our log distribution experiment 3c. Since only one MAG was created for this community, the MAG workflow was only able to detect SVs in the most abundant microbe. While rhea also decreases its detection ability with a decrease in coverage, it was able to detect 30% of SVs in a microbe with only 4x coverage.

Of the 125 SVs that were not detected by rhea in the even distribution, roughly 50% were not detected in the assembly graph, roughly 40% were in the graph but resolved into longer nodes rather than partaking in SV graph patterns, and the remaining 10% were called as the wrong SV type.

### 3.3 Cheese ripening temporal series

**Figure 4.**
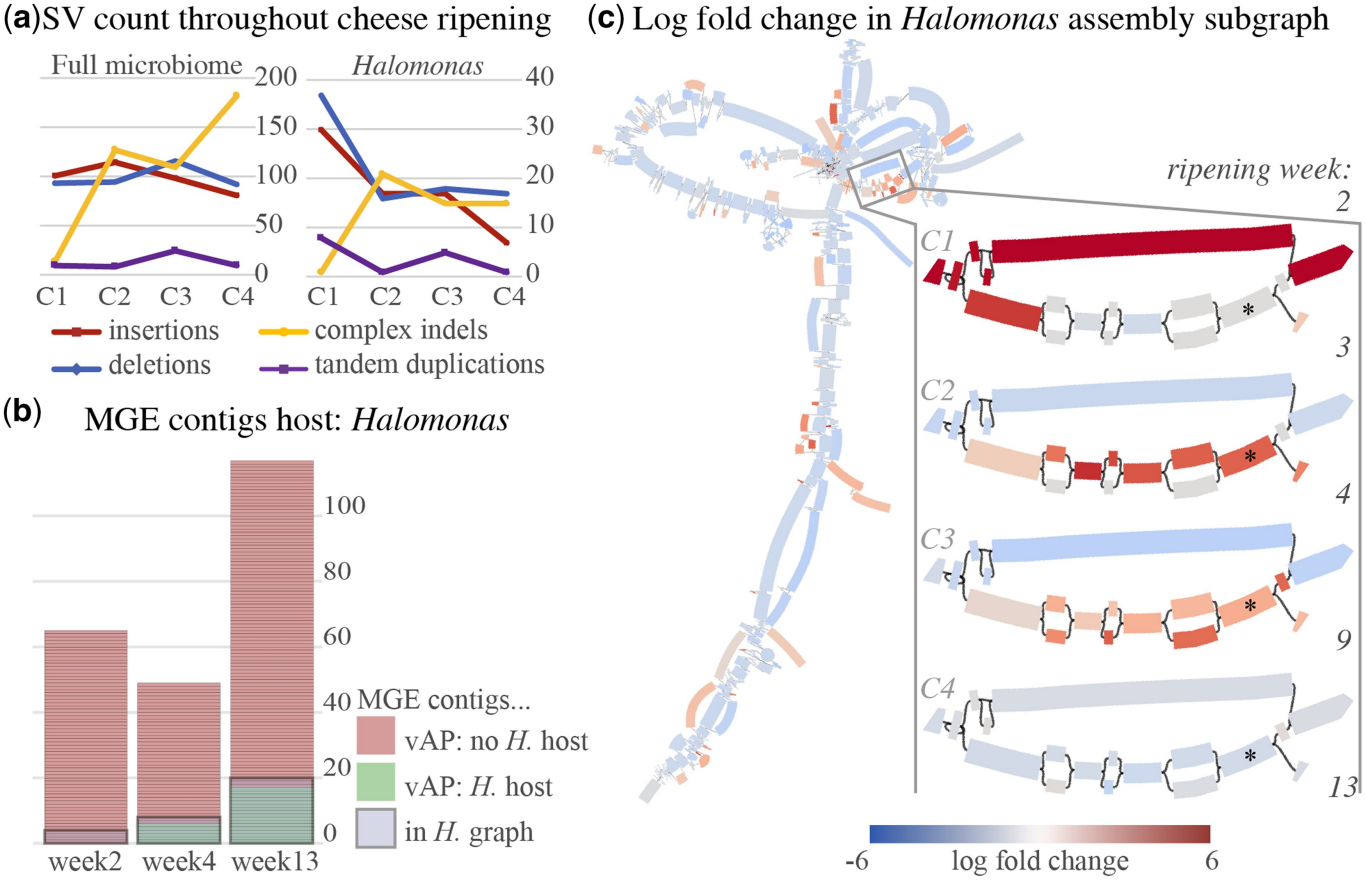
(a) SV counts detected by rhea for pairs of consecutive samples throughout cheese ripening (*C*1–4) for the entire community (Full microbiome) and exclusively the extracted *Halomonas* subgraph (*Halomonas*). (b) Plot where each stacked horizontal bar represents one of the labeled mobile genetic element (MGE) contigs, per the original cheese evaluation manuscript, for three sampling time points (week 2, 4, and 13). Each bar is colored to signify if viralAssociationPipeline (vAP) determined *Halomonas* as a host for that contig (green for yes; red for no). A grey box is drawn around a select stack for bars for each sample, signifying the MGE contigs that had a BLAST hit of >5% query coverage to our *Halomonas* subgraph. (c) Rhea and Bandage generated visual for the log fold change in coverage for the *Halomonas* subgraph. Left shows the complete *Halomonas* subgraph between weeks 4 and 9 (*C*3), selected for showing a general decrease in abundance yet an increase in abundance for several subsequences. In this graph visualization, each rectangle represents a sequence node. A line between two nodes *a* and *b* represents the presence of read overlap from either node *a* to node *b*, or vice versa. Each node is colored to show the change in coverage from week 4 to week 9, where a darker red represents an increase and darker blue for a decrease. Right zooms in on a small portion of the subgraph, selected due to one path showing favoritism over other paths over time, where the log fold change in coverage graph is shown for each pair of consecutive time points (*C*1–4). The graph node marked with a * indicates a sequence node containing the predicted type I restriction-modification system.

To demonstrate rhea’s ability to explore interesting microbial evolutionary patterns within a microbiome over time, PacBio HiFi metagenomic sequences taken from a cheese rind over the course of ripening were used as input (Saak *et al.* 2023). A total of five samples were included from sampling weeks 2, 3, 4, 9, and 13, creating four pairs of change (*C*1–4). Evaluating the assembly graph coverage visuals produced by rhea and Bandage (Wick *et al.* 2015), one connected component stood out for displaying significant graph complexity and diversity in coverage, implying a disproportionately large number of SVs. Rhea SV results indicated roughly 20% of SVs in the community to be contained in this subgraph (Fig. 4a). This connected component was then classified by GTDB-Tk under genus *Halomonas* and further exploration was pursued.

First, the ability for viral and plasmid mobile genetic elements (MGEs) to show signatures in the *Halomonas* subgraph was evaluated. In the original publication for the cheese samples, MGE contigs and putative hosts were established via Hi-C sequencing technology and overlap read coverage with the viralAssociatePipeline (Bickhart *et al.* 2019) for sampling weeks 2, 4, and 13. Their results showed *Halomonas* to be host for 0, 6, and 17 MGE contigs, respectively. A BLAST (Altschul *et al.* 1990) comparison of all MGE contigs against the *Halomonas* subgraph, showed all putative *Halomonas* MGE contigs to display signatures in our *Halomonas* subgraph (hit with more than 5% query coverage), despite previous host connections being defined via Hi-C sequencing and our graph being constructed solely on long-read sequences. An additional 4, 2, and 3 MGE contigs showed signature in the *Halomonas* subgraph without having a previous description of a *Halomonas* host for the time point for each of the three included sampling weeks, respectively (Fig. 4b), which may be false positives or novel host discovery. Finally, one noteworthy section of the *Halomonas* subgraph was selected for gene function analysis (Fig. 4c). Here, a newly emerged path (displayed lower option) shows an increase in coverage over time up until stabilizing by week 9, suggesting an evolutionary advantage over the alternative path (top option). Gene function predictions returned by SeqScreen (Balaji *et al.* 2023) showed the newly dominating path to contain a type I restriction-modification system that was not expressed in the alternative sequence. This suggests an evolutionary advantage due to phage protection in the *Halomonas* strains, which is unsurprising given the increasing number of phage interactions detected throughout ripening for *Halomonas*. Exploratory analysis here demonstrates an additional feature of rhea, which permitted the extraction of genomic subsequences that suggest an evolutionary advantage, gained insight into MGE hosts, and helped infer microbial interactions.

### 3.4 Hot spring microbial mat temperature series

**Figure 5.**
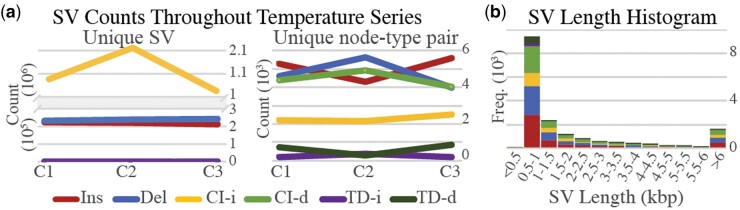
(a) SV counts detected by rhea for pairs of consecutive temperature gradient samples in increasing order (*C*1–3). The left “Unique SV” plot counts each unique SV as one, where the right “Unique node-type pair” plot counts each unique node-type pair as one (i.e. the same SV sequence labeled as an insertion between multiple different pairs of nodes is counted as one). The “Unique SV” plot contains a broken *y*-axis to improve visibility. In this way of counting, complex indel insertions (CI-i) and complex indel deletions (CI-d) contain the same values. The values for both tandem duplication categories, inserted duplicates (TD-i) and deleted duplicates (TD-d), are all under 1000 for both plots. (b) Histogram of the unique SV node-type pairs lengths colored by type, with overflow bin set at 6 kb.

To assess an environmental sample with complex interactions, rhea was run on a temperature series of samples taken from the Mushroom Spring microbial mat in Yellowstone National Park, USA. Samples were collected from four different portions of the mat with temperatures 50°C, 55°C, 60°C, and 65°C. Figure 5a displays the number of SVs reported and the number of unique SV sequence-type pairs observed between successive temperature increments (*C*1: 50°C to 55°C, *C*2: 55°C to 60°C, and *C*3: 60°C to 65°C). For insertions and deletions, the number of SVs detected is roughly three orders of magnitude greater than the number of unique SV sequence-type pairs. This implies that either the same SV sequence and type tend to occurs in many different genomic locations throughout the community or SVs are falsely inflated by rhea due to graph complexity and high-degree nodes (i.e. nodes that are either repeated in different locations or conserved across divergent strains). This pattern is also observed for complex indels, where SVs are counted to be roughly four orders of magnitude greater than unique SV sequence-type pairs. As for SV sequence length, the majority of reported SVs were between 500 and 1000 bp (Fig. 5b). Previous research closely analyzed two *Synechococcus* isolates from these mats and showed a large number of diverse insertion sequence (IS) activity occurring within the two strains (Nelson *et al.* 2011). Our findings suggest very high levels of transposon activity, gene exchange, and uncharacterized strains that occur in microbial mats. Further research is needed to confirm these findings and characterize the gene functions relevant to the SVs to provide additional insight into extremophile evolution and adaptation.

One sample (60°C) was selected to assess the read inclusion rate of alternative workflows for this community rife in unknown microbes. To evaluate a reference-based taxonomic classification method, reads were classified by Kraken2 with default database, where 42% of the reads were left unclassified. To evaluate a MAG creation workflow, MAGs were created with MetaFlye contigs and MetaBat2 binning, where roughly 30% of reads did not map to a binned contig. With rhea, 13.5% of reads did not align back to the constructed co-assembly graph.

### 3.5 Read to co-assembly graph mapping rates

To evaluate the ability for the constructed co-assembly graph to incorporate all sequenced reads, the percent of reads that did not align to the co-assembly graph were recorded. For the two ONT simulations, 8.1% and 8.4% of reads did not align to their co-assembly graph. However, when restricting only to those reads that mapped back to their reference (based on NanoSim reported error), only 0.2% and 0.4% did not align to their co-assembly graph. For the real datasets, the PacBio HiFi cheese reads showed few reads to not align (0.6%) while error-prone ONT hot spring reads had far more (13.5%).

### 3.6 Computational usage

Table 1 reports the CPU and RAM usage for rhea experimental results. All software analysis was completed on a Ubuntu 22.04 LTS system with 15 threads. The /usr/bin/time command was used to gather time and memory statistics. Reported CPU (central processing unit) time was calculated by summing the user and the system time; RAM (random access memory) requirements were determined using the maximum resident set size.

## 4 Discussion

Rhea is a graph-based method for detecting structural variants (SVs) between consecutive samples in long-read metagenome series data. Rhea avoids reference databases and MAGs by analyzing structural motifs and change in alignment coverage on a combined co-assembly graph for SV detection of intraspecies variations, lower abundance genomes, and novel organisms.

Long reads have been shown to improve the ability to detect SVs in isolate genomes (Ahsan *et al.* 2023). This led us to develop rhea for long reads, yet the fundamental idea could likely be expanded to short reads with further experimentation. Specifically, the type of co-assembly graph constructed should be evaluated since repeat assembly graphs are optimized for long reads (Kolmogorov *et al.* 2020). While our results did not show a strong correlation between the SV length and rhea’s ability to detect SVs (recall of 63% for SVs < 1000 bps in length, 62% for SVs 1000–1500 bps, and 83% for SVs >1500 bps), further evaluation is needed to determine if this holds true for a broader range of SV lengths.

One benefit of rhea is the inclusion of more reads into SV analysis than MAG- or reference-based approaches. When using low-error PacBio HiFi reads, we found less than 1% of the reads to get discarded due to an inability to align to the graph. In our simulated ONT reads, all reads that contained too many errors to be mapped back to the reference were discarded, while only <0.5% of remaining reads were discarded. We thus posit that the majority of unaligned reads are likely to be high-error reads, while the remainder may be from contamination or extremely low abundant organisms and SVs.

**Table 1.** CPU and RAM usage for rhea experiments.

	Reads	Base pairs	User+sys	RAM
Study	(million)	(billion)	time (h)	(GB)
HgtSIM (m0)	1.0	4.0	13	26
HgtSIM (m30)	1.0	4.0	13	26
ZymoBIOMICS	1.0	4.0	13	26
Cheese	1.8	23.1	154	47

Currently, rhea is only able to detect insertion, deletion, tandem duplication, and complex indel SV types between two metagenomes of similar microbes. Since these are detected through simple triangles and squares on the co-assembly graph, further development is required to permit detection of SVs over more complex regions of the graph and to reduce false positives of recurring SVs in graph regions with high-degree nodes. Detection could theoretically be expanded to inversions and translocations; however, we anticipate the need to maintain node directionality (whether the sequence is read forward or reverse) in the co-assembly graph. Rhea also decreases in its ability to detect SVs as the genome coverage decreases, and was unable to detect any SVs for genomes with less than 1x coverage. Further algorithm developments could help improve rhea for more sensitive detection in low abundance genomes.

While rhea has so far only been evaluated for SV detection over the course of microbiome series data, the idea of constructing a co-assembly graph and comparing the coverage between samples could be expanded beyond series data and used for different types of studies, such as cohort comparison analyses. However, caution should be taken with regards to the similarity of microbes across samples. Rhea detects SVs when reads from different samples align to similar areas within a co-assembly graph. As the communities diverge, graph alignment overlap between samples is expected to decrease. Further testing is needed to determine which divergence levels are too extreme for rhea’s algorithm. An additional consideration of cohort studies is the increased number of reads likely to be included in the co-assembly graph. As the graph may become too complex computationally, methods of downsampling sequences or alternate graph construction methods could be considered.

An additional benefit of rhea is that its results contain input data for the interactive visual software package Bandage (Wick *et al.* 2015) for exploration of changes in graph coverage throughout a metagenome series. This tool provides researchers with an efficient method to investigate sequence-level fluctuations while maintaining genome context, to ultimately extract sequences of interest as shown in Fig. 4c.

In lieu of metagenome-specific methods, metagenomes are often analyzed with methods and models developed for genomic analyses. Yet this simplification overlooks inherent complexities of dynamic and interdependent microbial ecosystems (Brito 2021). By viewing these communities holistically and acknowledging their intricate co-evolution with rhea, we can pinpoint microbial heterogeneity and evolution of these diverse and interconnected ecosystems.

## Supplementary Material

btae224_Supplementary_Data

## Data Availability

The data underlying this article are available as follows: 1) scripts, simulations, and complete results on OSF at https://osf.io, and can be accessed with project identifier FVHW8, 2) Rhea source code on GitHub at https://github.com/treangenlab/rhea, and 3) Hot spring long reads can be found on Sequence Read Archive (SRA) at https://www.ncbi.nlm.nih.gov/sra, under project PRJNA1072691.
