# Supplementary material for: Reference-free structural variant detection in microbiomes via long-read co-assembly graphs
Source: Bioinformatics. 2024 Jun 28;40(Suppl 1):i58–67. doi: 10.1093/bioinformatics/btae224 (PMC11211843; doi:10.1093/bioinformatics/btae224)
Supplement: btae224_Supplementary_Data [file btae224_supplementary_data.zip › btae224_Supplementary_Data/oup-authoring-template-doc.pdf]

User Manual
OUP Journals—Authoring template

Copyright Oxford University Press 2020

Prepared by SPi TEX Support

March 18, 2022

Contents

1 Introduction 2

2 Quick reference guide 2

3 How to start and prepare your article 3

4 Package features and some important settings 4
4.1 Language 4
4.2 Fonts 4

5 Preamble 4

6 Details on document class options 4

7 Major structures/elements 5

8 Front matter elements 5

9 Main matter elements 6
9.1 Section headings 6
9.2 Mathematical formulae 6
9.3 Figures and Tables 6
9.4 Lists 8
9.5 Theorem-like environments 9
9.6 Footnotes 9
9.7 Algorithms, Program codes, and Listings 9
9.8 Cross references 10
9.9 Cross citations 10

10 Back matter elements **10**
10.1 Appendices 10
10.2 References 11

11 Author biography **11**

12 Author supports **12**

13 Revision History **12**

1 Introduction

Oxford University Press has developed this authoring template to help authors in preparing articles. Authors are encouraged to use this template to produce LaTeX manuscripts which conform to OUP Journal styles. This document is a manual for authors to help using this template during article preparation. It features general guidelines and contains descriptions regarding various elements that can be used while preparing manuscripts. Authors are requested to refer to the file "readme" for details of files available for reference.

Please utilize the OUP authoring template to the maximum, rather than adding further packages or macros if possible. Please use semantic mark-up instead of formatting markup where there is a choice, for instance using `\emph` rather than `\it` or `\textit` to indicate emphasis.

This documentation is not intended to give an introduction to LaTeX. For questions concerning TeX systems/installations or the LaTeX mark-up language in general, please visit `http://tug.ctan.org/` or any other TeX user group worldwide. The essential reference for LaTeX is Mittelbach F., Goossens M. (2004) *The LaTeX Companion. 2nd edn.*, but there are many other good books about LaTeX.

2 Quick reference guide

- How to download and install - `https://miktex.org/download`
- Any system requirements - `https://miktex.org/kb/prerequisites-2-9`
- Quick steps to get started - `http://users.dickinson.edu/~richesod/latex/latexcheatsheet.pdf`
  `http://wch.github.io/latexsheet/latexsheet.pdf`
  `https://www.overleaf.com/learn`

3 How to start and prepare your article

It is assumed that you possess basic knowledge of LaTeX. Unless using LaTeX online (for example via Overleaf.com) ensure that you have LaTeX2e version installed on your computer. We suggest employing a recent TeXinstallation. You are provided with a class file "oup-authoring-template.cls". This template can be kept with your manuscript files. Note that the class file depends on the following packages which are standard and are available along with LaTeXinstallation:

graphicx	multirow	amsmath	amssymb	amsfonts
array	flushend	stfloats	color	xcolor
rotating	chngpage	totcount	fix-cm	algorithm
algorithmicx	algpseudocode	listings	url	crop

Apart from the above-listed packages, the following additional packages are used in the class file for providing add-on functionalities to the template:

subfloat	subfig	tikz	hyperref	footnote
mathrsfs	natbib	wrapfig	amsthm	apacite

To learn more about the underlying packages, please read the respective documentations (try, e.g., texdoc [package name] at your shell prompt or visit `http://tug.ctan.org/`).

You are given with sample tex file: oup-authoring-template.tex

Based on your journal style, we would suggest you to use the corresponding sample template file to start your manuscript preparation; other templates available in the package can be removed. Please save a copy of the template file before you start editing the file as per your requirement. These samples contain the lines for calling class files, the preamble area, and the start/end of document where major sample elements required for an article are placed. Comments are included for each element and are self-explanatory. You can add your actual manuscript content in place of these sample elements. The standard structure of each element for an article is explained in detail in the following sections.

To use the OUP authoring template, put all the package files in your working directory, edit the file "sample template file" in your preferred text editor, and run LaTeX as usual. The resulting layout is similar but not identical to the layout of the final article.

Please note that you are not responsible for any final page layout. It is not necessary (and is even sometimes undesirable) to do any fine-tuning with commands like `\break`, `\pagebreak`, `\vspace`, `\clearpage`, etc. Please use semantic mark-up as far as possible and avoid additional formatting commands.

4 Package features and some important settings

4.1 Language

English is the default language used for typesetting rules.

4.2 Fonts

Please refrain from using custom fonts.

Text fonts: Unlike the final published version, the authoring template uses non-commercial fonts: Computer Modern. These fonts are free version of the PostScript standard fonts and are supplied as part of all standard TEX distributions.

Math fonts: The standard Computer Modern math fonts are used.

5 Preamble

The preamble part comes between the document class line—`\documentclass{...}`—and the beginning of your document—`\begin{document}`. Use this preamble area to include additional packages and customized macros if any.

6 Details on document class options

a. Options available to select three different types of layout:
   - Modern
   - Traditional
   - Contemporary

b. Options available to select three different paper sizes:
   - Large
   - Medium
   - Small

c. namedate — for authoryear citation style; default reference citation style is numbered reference style

d. webpdf — for cropped paper size in the PDF output

e. unnumsec — to get unnumbered section heads

7 Major structures/elements

Article contents are divided into three main elementsfront matter, main matter, and back matter. The elements preceding the \maketitle tag are considered as front matter elements and the elements placed below the \maketitle tag are main matter elements. Elements found after the section “Conclusion” are considered as back matter elements.

**Front matter**	**Main matter**	**Back matter**
\title{...}	\section{...}	\begin{appendices}...\end{appendices}
\author{...}	\subsection{...}	\bibliographystyle{...}
\authormark{...}	\subsubsection{...}	\bibliography{...}
\address{...}	\paragraph{...}	\begin{biography}{...}{...}\end{biography}
\corresp[]{...}	\begin{algorithm}...\end{algorithm}	
\received{...}{...}{...}	\begin{lstlisting}...\end{lstlisting}	
\revised{...}{...}{...}	\begin{table}...\end{table}	
\accepted{...}{...}{...}	\begin{figure}...\end{figure}	
\abstract{...}	\begin{enumerate}...\end{enumerate}	
\keywords{...}	\begin{unlist}...\end{unlist}	
\boxedtext{...}	\begin{itemize}...\end{itemize}	
\editor{...}	\begin{theorem}...\end{theorem}	
\maketitle	\begin{proposition}...\end{proposition}	
	\begin{example}...\end{example}	
	\begin{definition}...\end{definition}	
	\begin{proof}...\end{proof}	
	\begin{equation}...\end{equation}	

8 Front matter elements

Required elements may vary depending on the journal to which you plan to submit. Check the instructions on the journals webpage carefully. The tagging details of article opener elements are as follows:

1. \title[<short-form-of-article-title>]{<article-title>}

   This tag contains two parameters, first one is optional and the second argument is mandatory. By default, the article title is printed as running heads on both odd/even pages. In case of lengthy article title, provide the short form of article title in the optional argument.

2. \author[<address-num>]{<author-name>}—to be used for the authors other than the corresponding author.

   \author[<address-num>,$\ast$]{<author-name>}—to be used for the corresponding author who is nominated as being responsible for the manuscript as it moves through the entire publication process. He is the time keeper during each phase of the publication process and the primary contact between the journal and all the other authors of the paper.

3. \address[<sequence>]{<address-details>}—affiliation/address details are provided inside this tag. In case of multiple addresses, just provide sequential Arabic numerals in the optional argument of this tag. This

numeral is used to denote the affiliation for the respective authors. In case of single author/address, this optional argument can be ignored. For example, refer below:

`\author{...}`

`\address{...}`

4. `\corresp[$\ast$]{...}` provide corresponding author's email id inside this tag.

5. The other tags listed below are self-explanatory. Note the 'received', 'revised' and 'accepted' dates are placeholders for the final publication: these can be left blank:

`\orgdiv{. . .}`	`\orgname{. . .}`	`\orgaddress{. . .}`	`\country{. . .}`	`\postcode{. . .}`
`\street{. . .}`	`\city{. . .}`	`\state{. . .}`	`\abstract{. . .}`	`\keywords{. . .}`
`\received{. . .}`	`\revised{. . .}`	`\accepted{. . .}`		

6. `\ORCID{...}`– provide ORCID link with logo inside this tag.

7. `\maketitle`—this tag is mandatory to print the front matter elements in the output.

9 Main matter elements

9.1 Section headings

The template allows four levels of headings in different styles:

```
\section{<First level heading>}
\subsection{<Second level heading>}
\subsubsection{<Third level heading>}
\paragraph{<Fourth level heading>}
```

To get unnumbered level heads, provide "unnumsec" option to `\documentclass[unnumsec]{oup-authoring-template}`.

9.2 Mathematical formulae

As the "amsmath" package provides various features for displayed equations and other mathematical constructs and you are strongly encouraged to use the mark-ups provided by this package. If possible, avoid using manual skips to align an equation.

9.3 Figures and Tables

The standard interface for graphic inclusion is the `\includegraphics` command provided by the graphicx package. The "draft" option provided by this package locally switches to draft mode, i.e. does not include the graphic, but leaves the correct space, and prints the filename. This option may be used to save

the processing time during compilation. Note that the `\graphicspath` command allows you to declare one or more folders where the graphicx package looks for the image files; hence, it is not necessary to write the path into each `\includegraphics` command.

The format used for numbered "figures/tables" is similar to the basic LaTeX format:

```
\begin{figure}[t]
\centering
\includegraphics{<image-file-name>}
\caption{<figure caption text>}\label{...}
\end{figure}
```

In case of double column layout, the above format puts figure captions/images to a single column width. To get spanned images, we need to provide the environment `\begin{figure*}...\end{figure*}`.

For the purpose of the sample, we have included the width of images in the optional argument of the `\includegraphics` tag. Please ignore this.

Images exceeding the text width should be set as rotated images. For this, we need to use `\begin{sidewaysfigure}...\end{sidewaysfigure}` instead of the `\begin{figure}...\end{figure}` environment. In case of double column layout, this format puts figure captions/images to single column width. To get spanned rotated images, use
`\begin{sidewaysfigure*}...\end{sidewaysfigure*}`.

If the journal requires that you include a graphical abstract, include this as an unnumbered figure directly after the text abstract with no caption, but adding the `\caption` package to the preamble and including the image as follows (note the file name for the graphical abstract):

```
\begin{figure}[t]
\centering
\includegraphics{graphical_abstract.jpg}
\captionsetup{labelformat=empty}
\caption{}
\label{...}
\end{figure}
```

The format for table is as follows:

```
\begin{table}[<float-position>]
\begin{center}
\begin{minipage}{<specify-table-width>}
\caption{...}\label{<table-label>}
\begin{tabular}{<column-alignment-preamble>}
\toprule
...  & ... & ... & ... \\
\midrule
```

```
... & ... & ... & ...\footnotemark{1} \\
... & ... & ... & ... \\
... & ... & ... & ...\footnotemark{2} \\
\botrule
\end{tabular}
\footnotetext{...}
\footnotetext[1]{...}
\footnotetext[2]{...}
\end{minipage}
\end{center}
\end{table}
```

Command to be used for rotated tables:

`\begin{sidewaystable}...\end{sidewaystable}`

To span tables across columns in double column layout:

`\begin{table*}...\end{table*}`

To span rotated tables across columns in double column layout:

`\begin{sidewaystable*}...\end{sidewaystable*}`

9.4 Lists

The default list commands available in LaTeX can be used to set different types of lists:

1. numbered: `\begin{enumerate}...\end{enumerate}`

   first level Arabic numerals;

   second level lowercase alphabet;

   third level lowercase roman numerals;

2. unnumbered: `\begin{unlist}...\end{unlist}`

3. custom list: `\begin{itemize}...\end{itemize}`

   first level bulleted;

   second level bulleted.

Nested lists are allowed for numbered and custom lists.

9.5 Theorem-like environments

For theorem-like environments, we require the "amsthm" package. There are three types of predefined theorem styles: thmstyleone, thmstyletwo, and thmstylethree. The below table shows the output details for each style:

thmstyleone	Numbered, theorem head in bold font and theorem text in italic style
thmstyletwo	Numbered, theorem head in italic font and theorem text in roman style
thmstylethree	Numbered, theorem head in bold font and theorem text in roman style

As per the output requirement, the corresponding new theorem style should be defined in the preamble area. For example, if you require a "Proposition" environment to be set with "thmstyletwo," then we need to include the below lines in the preamble area:

```
\theoremstyle{thmstyletwo}
\newtheorem{proposition}{Proposition}
```

Refer to the "amsthm" package documentation for more details about the additional features available for new theorem styles. As well as the above, a predefined "proof" environment is available. This environment prints the "Proof" head in italic style and "body text" in Roman style with an open square at the end of each proof environment.

```
\begin{proof}...\end{proof}
```

9.6 Footnotes

Footnotes are produced with the standard LaTeX command `\footnote{<Some text>}`. This typesets a numerical flag at the location of the footnote command and places the footnote text at the bottom of the page.

9.7 Algorithms, Program codes, and Listings

The packages "algorithm," "algorithmicx," and "algpseudocode" are used for setting algorithms in LaTeX. For algorithms, use the below format:

```
\begin{algorithm}
\caption{<alg-caption>}\label{<alg-label>}
\begin{algorithmic}[1]
...
\end{algorithmic}
\end{algorithm}
```

Refer the above-listed package documentation for more details before setting algorithms.

To set program codes, the “program” package is required and the command to be used is `\begin{program}...\end{program}`.

The command `\begin{lstlisting}...\end{lstlisting}` is used to set “verbatim” like environments. Refer to the ”lstlisting” package documentation for more details.

9.8 Cross references

LaTeX is able to automatically insert hypertext links within a document:

- the `\ref{}` command adds a clickable link to the referred object;
- the `\label{}` command automatically inserts a target;

9.9 Cross citations

To make a citation in the text, use `\citep{...}` for a parenthetical citation (Jones et al., 1990), `\citet{...}` for a textual one, as Jones et al. (1990). Both `\citep` and `\citet` are defined by “natbib” and are thus not standard. The standard LaTeX command `\cite` should be avoided, because it behaves like `\citet` for authoryear citations, but like `\citep` for numerical ones. There also exist the starred versions `\citet*` and `\citep*` that print the full author list, and not just the abbreviated one. All of these may take one or two optional arguments to add some text before and after the citation:

\citet{jon90}	Jones et al. (1990)
\citet[chap.~2]{jon90}	Jones et al. (1990, chap. 2)
\citep{jon90}	(Jones et al., 1990)
\citep[chap.~2]{jon90}	(Jones et al., 1990, chap. 2)
\citep[see][]{jon90}	(see Jones et al., 1990)
\citep[see][chap.~2]{jon90}	(see Jones et al., 1990, chap. 2)
\citet*{jon90}	Jones, Baker, and Williams (1990)
\citep*{jon90}	(Jones, Baker, and Williams, 1990)

Please refer to the “natbib” package documentation for guidance on other citation commands.

10 Back matter elements

10.1 Appendices

This section is set with a `\begin{appendices}...\end{appendices}` environment. All the other commands used to set section heads, tables, and figures inside this section remain the same as the main body text.

10.2 References

The basic bibliography environment is accepted for setting a reference section:

```
\begin{thebibliography}{9}
\bibitem{bib1} ...
\bibitem{bib2} ...
\end{thebibliography}
```

However, BiBTEX is the preferred format for references. BiBTEX automates most of the work involved in references for articles. Using BiBTEX options, both citations and references can be automatically updated to the preferred reference style. That is, you need not apply reference style tags for each element manually; it promotes structured writing. Basically, BiBTEX works with two parts of the references: *content* and *style*. The *content* is stored separately in a plain text database file called .bib, in which each entry is structured to distingiush diferent types of entries and fields. The *style* and presentation of the database content are processed with the help of BiBTEX program using a style file called .bst (bibliography style file).

You are requested to use the sample bib file "reference.bib" provided as a base for preparing your own .bib file. For the "author-year" citation style, use "abbrvnat" bst and for "numbered" citation style use "plain" bst, by adding the following instructions to the preamble.

1. author-year citation style = `\bibliographystyle{abbrvnat}`
2. numbered citation style = `\bibliographystyle{unsrt}`

Then include your .bib file at the end of your document as shown below:

```
\bibliography{<bib-file-without-extension>}
```

To generate the .bbl file, you should only need to compile LATEX/PDFLATEXonce, then BIBTEX, then LATEX/PDFLATEXtwice more. The resulting bibliography is ready for typesetting with all formatting tags rendered according to the chosen reference style. For more details, please visit `http://www.bibtex.org`.

11 Author biography

If this section is required by the journal, please use the below command:

```
\begin{biography}
{\includegraphics{<image-file>}}
{\author{<author-Name>} biography-text}
\end{biography}
```

12 Author supports

General support for LaTeX related questions can be obtained from the Internet newsgroup comp.text.tex. Frequently asked questions are available in various web sites dealing with LaTeX.

13 Revision History

Revision State	Revision Date	Version Number	Revision History
00	16 March, 2020	Version 1.0	
01	18 March, 2022	Version 1.1	
