# Supplementary material for: Reference-free structural variant detection in microbiomes via long-read co-assembly graphs
Source: Bioinformatics. 2024 Jun 28;40(Suppl 1):i58–67. doi: 10.1093/bioinformatics/btae224 (PMC11211843; doi:10.1093/bioinformatics/btae224)
Supplement: btae224_Supplementary_Data [file btae224_supplementary_data.zip › btae224_Supplementary_Data/oup-authoring-template.pdf]

Journal Title Here, 2022, pp. 1–9

doi: DOI HERE
Advance Access Publication Date: Day Month Year
Paper

PAPER

Article Title

First Author,[1,*] Second Author,[2] Third Author,[3] Fourth Author[3] and Fifth Author[4]

[1]Department, Organization, Street, Postcode, State, Country, [2]Department, Organization, Street, Postcode, State, Country, [3]Department, Organization, Street, Postcode, State, Country and [4]Department, Organization, Street, Postcode, State, Country
[*]Corresponding author. email-id.com
FOR PUBLISHER ONLY Received on Date Month Year; revised on Date Month Year; accepted on Date Month Year

Abstract

Abstracts must be able to stand alone and so cannot contain citations to the paper's references, equations, etc. An abstract must consist of a single paragraph and be concise. Because of online formatting, abstracts must appear as plain as possible.

Key words: keyword1, Keyword2, Keyword3, Keyword4

Introduction

The introduction introduces the context and summarizes the manuscript. It is importantly to clearly state the contributions of this piece of work. Lorem ipsum dolor sit amet, consectetur adipiscing elit, sed do eiusmod tempor incididunt ut labore et dolore magna aliqua. Ut enim ad minim veniam, quis nostrud exercitation ullamco laboris nisi ut aliquip ex ea commodo consequat. Duis aute irure dolor in reprehenderit in voluptate velit esse cillum dolore eu fugiat nulla pariatur. Excepteur sint occaecat cupidatat non proident, sunt in culpa qui officia deserunt mollit anim id est laborum.

This is an example of a new parapgraph with a numbered footnote[1] and a second footnote marker.[2]

This is an example for first level head - section head

Lorem ipsum dolor sit amet, consectetur adipiscing elit, sed do eiusmod tempor incididunt ut labore et dolore magna aliqua. Ut enim ad minim veniam, quis nostrud exercitation ullamco laboris nisi ut aliquip ex ea commodo consequat. Duis aute irure dolor in reprehenderit in voluptate velit esse cillum dolore eu fugiat nulla pariatur. Excepteur sint occaecat cupidatat non proident, sunt in culpa qui officia deserunt mollit anim id est laborum (refer Section 5).

This is an example for second level head - subsection head

Lorem ipsum dolor sit amet, consectetur adipiscing elit, sed do eiusmod tempor incididunt ut labore et dolore magna aliqua. Ut enim ad minim veniam, quis nostrud exercitation ullamco laboris nisi ut aliquip ex ea commodo consequat. Duis aute irure dolor in reprehenderit in voluptate velit esse cillum dolore eu fugiat nulla pariatur. Excepteur sint occaecat cupidatat non proident, sunt in culpa qui officia deserunt mollit anim id est laborum.

This is an example for third level head - subsubsection head

Lorem ipsum dolor sit amet, consectetur adipiscing elit, sed do eiusmod tempor incididunt ut labore et dolore magna aliqua. Ut enim ad minim veniam, quis nostrud exercitation ullamco laboris nisi ut aliquip ex ea commodo consequat.

This is an example for fourth level head - paragraph head
Lorem ipsum dolor sit amet, consectetur adipiscing elit, sed do eiusmod tempor incididunt ut labore et dolore magna aliqua. Ut enim ad minim veniam, quis nostrud exercitation ullamco laboris nisi ut aliquip ex ea commodo consequat. Duis aute irure dolor in reprehenderit in voluptate velit esse cillum dolore eu fugiat nulla pariatur. Excepteur sint occaecat cupidatat non proident, sunt in culpa qui officia deserunt mollit anim id est laborum.

This is an example for first level head

This is an example for second level head - subsection head

This is an example for third level head - subsubsection head

Lorem ipsum dolor sit amet, consectetur adipiscing elit, sed do eiusmod tempor incididunt ut labore et dolore magna aliqua. Ut enim ad minim veniam, quis nostrud exercitation ullamco laboris nisi ut aliquip ex ea commodo consequat. Duis aute irure dolor in reprehenderit in voluptate velit esse cillum dolore eu fugiat nulla pariatur. Excepteur sint occaecat cupidatat non

[1] https://data.gov.uk/

[2] Example of footnote text.

© The Author 2022. Published by Oxford University Press. All rights reserved. For permissions, please e-mail: journals.permissions@oup.com

proident, sunt in culpa qui officia deserunt mollit anim id est laborum.

This is an example for fourth level head - paragraph head
Lorem ipsum dolor sit amet, consectetur adipiscing elit, sed do eiusmod tempor incididunt ut labore et dolore magna aliqua. Ut enim ad minim veniam, quis nostrud exercitation ullamco laboris nisi ut aliquip ex ea commodo consequat. Duis aute irure dolor in reprehenderit in voluptate velit esse cillum dolore eu fugiat nulla pariatur. Excepteur sint occaecat cupidatat non proident, sunt in culpa qui officia deserunt mollit anim id est laborum.

Equations

Equations in LaTeX can either be inline or set as display equations. For inline equations use the `$...$` commands. Eg: the equation $H\psi = E\psi$ is written via the command `$H \psi = E \psi$`.

For display equations (with auto generated equation numbers) one can use the equation or eqnarray environments:

$$\|\tilde{X}(k)\|^2 \leq \frac{\sum\limits_{i=1}^{p} \left\|\tilde{Y}_i(k)\right\|^2 + \sum\limits_{j=1}^{q} \left\|\tilde{Z}_j(k)\right\|^2}{p+q}, \tag{1}$$

where,

$$D_\mu = \partial_\mu - ig\frac{\lambda^a}{2}A^a_\mu$$
$$F^a_{\mu\nu} = \partial_\mu A^a_\nu - \partial_\nu A^a_\mu + gf^{abc}A^b_\mu A^a_\nu. \tag{2}$$

Notice the use of `\nonumber` in the align environment at the end of each line, except the last, so as not to produce equation numbers on lines where no equation numbers are required. The `\label{}` command should only be used at the last line of an align environment where `\nonumber` is not used.

$$Y_\infty = \left(\frac{m}{\text{GeV}}\right)^{-3}\left[1 + \frac{3\ln(m/\text{GeV})}{15} + \frac{\ln(c_2/5)}{15}\right]. \tag{3}$$

The class file also supports the use of `\mathbb{}`, `\mathscr{}` and `\mathcal{}` commands. As such `\mathbb{R}`, `\mathscr{R}` and `\mathcal{R}` produces $\mathbb{R}$, $\mathscr{R}$ and $\mathcal{R}$ respectively (refer Subsubsection A.1.1).

Lorem ipsum dolor sit amet, consectetur adipiscing elit, sed do eiusmod tempor incididunt ut labore et dolore magna aliqua. Ut enim ad minim veniam, quis nostrud exercitation ullamco laboris nisi ut aliquip ex ea commodo consequat. Duis aute irure dolor in reprehenderit in voluptate velit esse cillum dolore eu fugiat nulla pariatur. Excepteur sint occaecat cupidatat non proident, sunt in culpa qui officia deserunt mollit anim id est laborum. Lorem ipsum dolor sit amet, consectetur adipiscing elit, sed do eiusmod tempor incididunt ut labore et dolore magna aliqua. Ut enim ad minim veniam, quis nostrud exercitation ullamco laboris nisi ut aliquip ex ea commodo consequat. Duis aute irure dolor in reprehenderit in voluptate velit esse cillum dolore eu fugiat nulla pariatur. Excepteur sint occaecat cupidatat non proident, sunt in culpa qui officia deserunt mollit anim id est laborum. Lorem ipsum dolor sit amet, consectetur adipiscing elit, sed do eiusmod tempor incididunt ut labore et dolore magna aliqua. Ut enim ad minim veniam, quis nostrud exercitation ullamco laboris nisi ut aliquip ex ea commodo consequat.

Table 1. Caption text

column 1	column 2	column 3	column 4
row 1	data 1	data 2	data 3
row 2	data 4	data 5[1]	data 6
row 3	data 7	data 8	data 9[2]

Source: This is an example of table footnote this is an example of table footnote this is an example of table footnote this is an example of table footnote this is an example of table footnote

[1]Example for a first table footnote.

[2]Example for a second table footnote.

Tables

Tables can be inserted via the normal table and tabular environment. To put footnotes inside tables one has to Lorem ipsum dolor sit amet, consectetur adipiscing elit, sed do eiusmod tempor incididunt ut labore et dolore magna aliqua. Ut enim ad minim veniam, quis nostrud exercitation ullamco laboris nisi ut aliquip ex ea commodo consequat. Duis aute irure dolor in reprehenderit in voluptate velit esse cillum dolore eu fugiat nulla pariatur. Excepteur sint occaecat cupidatat non proident, sunt in culpa qui officia deserunt mollit anim id est laborum. use the additional "tablenotes" environment enclosing the tabular environment. The footnote appears just below the table itself (refer Tables 1 and 2).

```
\begin{table}[t]
\begin{center}
\begin{minipage}{<width>}
\caption{<table-caption>\label{<table-label>}}%
\begin{tabular}{@{}llll@{}}
\toprule
column 1 & column 2 & column 3 & column 4\\
\midrule
row 1 & data 1 & data 2          & data 3 \\
row 2 & data 4 & data 5$^{1}$ & data 6 \\
row 3 & data 7 & data 8      & data 9$^{2}$\\
\botrule
\end{tabular}
\begin{tablenotes}%
\item Source: Example for source.
\item[$^{1}$] Example for a 1st table footnote.
\item[$^{2}$] Example for a 2nd table footnote.
\end{tablenotes}
\end{minipage}
\end{center}
\end{table}
```

Lengthy tables which do not fit within textwidth should be set as rotated tables. For this, we need to use `\begin{sidewaystable}... \end{sidewaystable}` instead of `\begin{table}... \end{table}` environment.

Figures

As per display LaTeX standards one has to use eps images for `latex` compilation and `pdf/jpg/png` images for `pdflatex` compilation. This is one of the major differences between `latex` and `pdflatex`. The images should be single-page documents. The command for inserting images for `latex` and `pdflatex` can be generalized. The package used to insert images in

Table 2. Example of a lengthy table which is set to full textwidth.

	Element 1[1]			Element 2[2]		
Project	Energy	σ_{calc}	σ_{expt}	Energy	σ_{calc}	σ_{expt}
Element 3	990 A	1168	1547 ± 12	780 A	1166	1239 ± 100
Element 4	500 A	961	922 ± 10	900 A	1268	1092 ± 40

Note: This is an example of table footnote this is an example of table footnote this is an example of table footnote this is an example of table footnote this is an example of table footnote

[1]Example for a first table footnote.

[2]Example for a second table footnote.

Fig. 1. This is a widefig. This is an example of a long caption this is an example of a long caption this is an example of a long caption this is an example of a long caption

`latex/pdflatex` is the graphicx package. Figures can be inserted via the normal figure environment as shown in the below example:

```
\begin{figure}[t]
        \centering\includegraphics{<eps-file>}
        \caption{<figure-caption>}
        \label{<figure-label>}
\end{figure}
```

Test text here.

For sample purposes, we have included the width of images in the optional argument of `\includegraphics` tag. Please ignore this. Lengthy figures which do not fit within textwidth should be set in rotated mode. For rotated figures, we need to use `\begin{sidewaysfigure}` ... `\end{sidewaysfigure}` instead of the `\begin{figure}` ... `\end{figure}` environment.

Algorithms, Program codes and Listings

Packages `algorithm`, `algorithmicx` and `algpseudocode` are used for setting algorithms in latex. For this, one has to use the below format:

```
\begin{algorithm}
\caption{<alg-caption>}\label{<alg-label>}
\begin{algorithmic}[1]
. . .
\end{algorithmic}
\end{algorithm}
```

You may need to refer to the above-listed package documentations for more details before setting an `algorithm` environment. To set program codes, one has to use the `program` package. We need to use the `\begin{program}` ... `\end{program}` environment to set program codes.

Similarly, for `listings`, one has to use the `listings` package. The `\begin{lstlisting}` ... `\end{lstlisting}` environment is used to set environments similar to the `verbatim` environment. Refer to the `lstlisting` package documentation for more details on this.

Algorithm 1 Calculate $y = x^n$

Require: $n \geq 0 \vee x \neq 0$
Ensure: $y = x^n$

```
y ⇐ 1
if n < 0 then
    X ⇐ 1/x
    N ⇐ −n
else
    X ⇐ x
    N ⇐ n
end if
while N ≠ 0 do
    if N is even then
        X ⇐ X × X
        N ⇐ N/2
    else[N is odd]
        y ⇐ y × X
        N ⇐ N − 1
    end if
end while
```

```
begin
{ do nothing }
end;
Write('Case insensitive ');
Write('Pascal keywords.');
```

Cross referencing

Environments such as figure, table, equation, and align can have a label declared via the `\label{#label}` command. For figures and table environments one should use the `\label{}` command inside or just below the `\caption{}` command. One can then use the `\ref{#label}` command to cross-reference them. As an example, consider the label declared for Figure 1 which is `\label{fig1}`. To cross-reference it, use the command `Figure \ref{fig1}`, for which it comes up as “Figure 1”.

Details on reference citations

With standard numerical .bst files, only numerical citations are possible. With an author-year .bst file, both numerical and author-year citations are possible.

If author-year citations are selected, `\bibitem` must have one of the following forms:

```
\bibitem[Jones et al.(1990)]{key}...
```

Fig. 2. This is a widefig. This is an example of a long caption this is an example of a long caption this is an example of a long caption this is an example of a long caption

```
\bibitem[Jones et al.(1990)Jones,
                 Baker, and Williams]{key}...
\bibitem[Jones et al., 1990]{key}...
\bibitem[\protect\citeauthoryear{Jones,
                 Baker, and Williams}
                 {Jones et al.}{1990}]{key}...
\bibitem[\protect\citeauthoryear{Jones et al.}
                 {1990}]{key}...
\bibitem[\protect\astroncite{Jones et al.}
                 {1990}]{key}...
\bibitem[\protect\citename{Jones et al., }
                 1990]{key}...
\harvarditem[Jones et al.]{Jones, Baker, and
                 Williams}{1990}{key}...
```

This is either to be made up manually, or to be generated by an appropriate .bst file with BibTeX. Then,

```
                    Author-year mode
                        || Numerical mode
\citet{key} ==>>  Jones et al. (1990)
                        || Jones et al. [21]
\citep{key} ==>> (Jones et al., 1990) || [21]
```

Multiple citations as normal:

```
\citep{key1,key2} ==> (Jones et al., 1990;
                         Smith, 1989)||[21,24]
        or (Jones et al., 1990, 1991)||[21,24]
        or (Jones et al., 1990a,b)   ||[21,24]
```

`\cite{key}` is the equivalent of `\citet{key}` in author-year mode and of `\citep{key}` in numerical mode. Full author lists may be forced with `\citet*` or `\citep*`, e.g.

```
\citep*{key} ==>> (Jones, Baker, and Mark, 1990)
```

Optional notes as:

```
\citep[chap. 2]{key}     ==>>
        (Jones et al., 1990, chap. 2)
\citep[e.g.,][]{key}     ==>>
        (e.g., Jones et al., 1990)
\citep[see][pg. 34]{key} ==>>
        (see Jones et al., 1990, pg. 34)
```

(Note: in standard LaTeX, only one note is allowed, after the ref. Here, one note is like the standard, two make pre- and post-notes.)

```
\citealt{key}   ==>> Jones et al. 1990
\citealt*{key}  ==>> Jones, Baker, and
                        Williams 1990
\citealp{key}   ==>> Jones et al., 1990
\citealp*{key}  ==>> Jones, Baker, and
                        Williams, 1990
```

Additional citation possibilities (both author-year and numerical modes):

```
\citeauthor{key}       ==>> Jones et al.
\citeauthor*{key}      ==>> Jones, Baker, and
                                Williams
\citeyear{key}         ==>> 1990
\citeyearpar{key}      ==>> (1990)
\citetext{priv. comm.} ==>> (priv. comm.)
\citenum{key}          ==>> 11 [non-superscripted]
```

Note: full author lists depend on whether the bib style supports them; if not, the abbreviated list is printed even when full is requested.
For names like della Robbia at the start of a sentence, use

```
\Citet{dRob98}      ==>> Della Robbia (1998)
\Citep{dRob98}      ==>> (Della Robbia, 1998)
\Citeauthor{dRob98} ==>> Della Robbia
```

The following is an example for `\cite{...}`: Rahman and Abdul (2019). Another example for `\citep{...}`: (Bahdanau et al., 2014; Imboden et al., 2018; Motiian et al., 2017; Murphy, 2012; Ji et al., 2012). Sample cites here Krizhevsky et al. (2012); Horvath and Raj (2018) and Pyrkov et al. (2018), Wang et al. (2018), LeCun et al. (2015); Zhang et al. (2018); Ravì et al. (2016).

Lists

List in LaTeX can be of three types: numbered, bulleted and unnumbered. The "enumerate" environment produces a numbered list, the "itemize" environment produces a bulleted list and the "unlist" environment produces an unnumbered list. In each environment, a new entry is added via the `\item` command.

1. This is the 1st item
2. Enumerate creates numbered lists, itemize creates bulleted lists and unnumerate creates unnumbered lists.

    a. Second level numbered list. Enumerate creates numbered lists, itemize creates bulleted lists and description creates unnumbered lists.
    b. Second level numbered list. Enumerate creates numbered lists, itemize creates bulleted lists and description creates unnumbered lists.

        (i) Third level numbered list. Enumerate creates numbered lists, itemize creates bulleted lists and description creates unnumbered lists.
        (ii) Third level numbered list. Enumerate creates numbered lists, itemize creates bulleted lists and description creates unnumbered lists.

Table 3. Tables which are too long to fit, should be written using the "sidewaystable" environment as shown here

Projectile	Element 1[1]			Element[2]		
	Energy	σ_{calc}	σ_{expt}	Energy	σ_{calc}	σ_{expt}
Element 3	990 A	1168	1547 ± 12	780 A	1166	1239 ± 100
Element 4	500 A	961	922 ± 10	900 A	1268	1092 ± 40

Note: This is an example of a table footnote this is an example of a table footnote this is an example of a table footnote this is an example of a table footnote this is an example of a table footnote

[1]This is an example of a table footnote

Fig. 3. This is an example for a sideways figure. This is an example of a long caption this is an example of a long caption this is an example of a long caption this is an example of a long caption

    c. Second level numbered list. Enumerate creates numbered lists, itemize creates bulleted lists and description creates unnumbered lists.
    d. Second level numbered list. Enumerate creates numbered lists, itemize creates bulleted lists and description creates unnumbered lists.

3. Enumerate creates numbered lists, itemize creates bulleted lists and description creates unnumbered lists.
4. Numbered lists continue.

Lists in LaTeX can be of three types: enumerate, itemize and description. In each environment, a new entry is added via the `\item` command.

- First level bulleted list. This is the 1st item
- First level bulleted list. Itemize creates bulleted lists and description creates unnumbered lists.
    - Second level dashed list. Itemize creates bulleted lists and description creates unnumbered lists.
    - Second level dashed list. Itemize creates bulleted lists and description creates unnumbered lists.
    - Second level dashed list. Itemize creates bulleted lists and description creates unnumbered lists.
- First level bulleted list. Itemize creates bulleted lists and description creates unnumbered lists.
- First level bulleted list. Bullet lists continue.

Example for unnumbered list items:

Sample unnumberd list text. Sample unnumberd list text. Sample unnumberd list text. Sample unnumberd list text. Sample unnumberd list text.

Sample unnumberd list text. Sample unnumberd list text. Sample unnumberd list text.

sample unnumberd list text. Sample unnumberd list text. Sample unnumberd list text. Sample unnumberd list text. Sample unnumberd list text. Sample unnumberd list text. Sample unnumberd list text.

Examples for theorem-like environments

For theorem-like environments, we require the `amsthm` package. There are three types of predefined theorem styles - `thmstyleone`, `thmstyletwo` and `thmstylethree` (check your journal's instructions page in case a specific style is required).

`thmstyleone`	Numbered, theorem head in bold font and theorem text in italic style
`thmstyletwo`	Numbered, theorem head in roman font and theorem text in italic style
`thmstylethree`	Numbered, theorem head in bold font and theorem text in roman style

Theorem 1 (Theorem subhead) *Example theorem text. Example theorem text. Example theorem text. Example theorem text. Example theorem text. Example theorem text. Example theorem text. Example theorem text. Example theorem text. Example theorem text. Example theorem text.*

Quisque ullamcorper placerat ipsum. Cras nibh. Morbi vel justo vitae lacus tincidunt ultrices. Lorem ipsum dolor sit amet, consectetuer adipiscing elit. In hac habitasse platea dictumst. Integer tempus convallis augue.

Proposition 2 *Example proposition text. Example proposition text. Example proposition text. Example proposition text. Example proposition text. Example proposition text. Example proposition text. Example proposition text. Example proposition text. Example proposition text.*

Nulla malesuada porttitor diam. Donec felis erat, congue non, volutpat at, tincidunt tristique, libero. Vivamus viverra fermentum felis. Donec nonummy pellentesque ante.

Example 1 *Phasellus adipiscing semper elit. Proin fermentum massa ac quam. Sed diam turpis, molestie vitae, placerat a, molestie nec, leo. Maecenas lacinia. Nam ipsum ligula, eleifend at, accumsan nec, suscipit a, ipsum. Morbi blandit ligula feugiat magna. Nunc eleifend consequat lorem.*

Nulla malesuada porttitor diam. Donec felis erat, congue non, volutpat at, tincidunt tristique, libero. Vivamus viverra fermentum felis. Donec nonummy pellentesque ante.

Remark 1 *Phasellus adipiscing semper elit. Proin fermentum massa ac quam. Sed diam turpis, molestie vitae, placerat a, molestie nec, leo. Maecenas lacinia. Nam ipsum ligula, eleifend at, accumsan nec, suscipit a, ipsum. Morbi blandit ligula feugiat magna. Nunc eleifend consequat lorem.*

Quisque ullamcorper placerat ipsum. Cras nibh. Morbi vel justo vitae lacus tincidunt ultrices. Lorem ipsum dolor sit amet, consectetuer adipiscing elit. In hac habitasse platea dictumst.

Definition 1 (Definition sub head) Example definition text. Example definition text. Example definition text. Example definition text. Example definition text. Example definition text. Example definition text. Example definition text.

Apart from the above styles, we have the `\begin{proof} ... \end{proof}` environment - with the proof head in italic style and the body text in roman font with an open square at the end of each proof environment.

Proof Example for proof text. Example for proof text. Example for proof text. Example for proof text. Example for proof text. Example for proof text. Example for proof text. Example for proof text. Example for proof text. Example for proof text. □

Nam dui ligula, fringilla a, euismod sodales, sollicitudin vel, wisi. Morbi auctor lorem non justo. Nam lacus libero, pretium at, lobortis vitae, ultricies et, tellus. Donec aliquet, tortor sed accumsan bibendum, erat ligula aliquet magna, vitae ornare odio metus a mi.

Proof of Theorem 1 Example for proof text. Example for proof text. Example for proof text. Example for proof text. Example for proof text. Example for proof text. Example for proof text. Example for proof text. Example for proof text. Example for proof text. □

For a quote environment, one has to use `\begin{quote}...\end{quote}`

> Quoted text example. Aliquam porttitor quam a lacus. Praesent vel arcu ut tortor cursus volutpat. In vitae pede quis diam bibendum placerat. Fusce elementum convallis neque. Sed dolor orci, scelerisque ac, dapibus nec, ultricies ut, mi. Duis nec dui quis leo sagittis commodo.

Donec congue. Maecenas urna mi, suscipit in, placerat ut, vestibulum ut, massa. Fusce ultrices nulla et nisl (refer Figure 3). Pellentesque habitant morbi tristique senectus et netus et malesuada fames ac turpis egestas. Etiam ligula arcu, elementum a, venenatis quis, sollicitudin sed, metus. Donec nunc pede, tincidunt in, venenatis vitae, faucibus vel (refer Table 3).

Conclusion

Some Conclusions here.

Section title of first appendix

Nam dui ligula, fringilla a, euismod sodales, sollicitudin vel, wisi. Morbi auctor lorem non justo. Nam lacus libero, pretium at, lobortis vitae, ultricies et, tellus. Donec aliquet, tortor sed accumsan bibendum, erat ligula aliquet magna, vitae ornare odio metus a mi. Morbi ac orci et nisl hendrerit mollis. Suspendisse ut massa. Cras nec ante. Pellentesque a nulla. Cum sociis natoque penatibus et magnis dis parturient montes, nascetur ridiculus mus. Aliquam tincidunt urna. Nulla ullamcorper vestibulum turpis. Pellentesque cursus luctus mauris.

Subsection title of first appendix

Nam dui ligula, fringilla a, euismod sodales, sollicitudin vel, wisi. Morbi auctor lorem non justo. Nam lacus libero, pretium at, lobortis vitae, ultricies et, tellus. Donec aliquet, tortor sed accumsan bibendum, erat ligula aliquet magna, vitae ornare odio metus a mi. Morbi ac orci et nisl hendrerit mollis. Suspendisse ut massa. Cras nec ante. Pellentesque a nulla. Cum sociis natoque penatibus et magnis dis parturient montes, nascetur ridiculus mus. Aliquam tincidunt urna. Nulla ullamcorper vestibulum turpis. Pellentesque cursus luctus mauris.

Subsubsection title of first appendix

Example for an unnumbered figure:

Fusce mauris. Vestibulum luctus nibh at lectus. Sed bibendum, nulla a faucibus semper, leo velit ultricies tellus, ac venenatis arcu wisi vel nisl. Vestibulum diam. Aliquam pellentesque, augue quis sagittis posuere, turpis lacus congue quam, in hendrerit risus eros eget felis.

Table 4. This is an example of Appendix table showing food requirements of army, navy and airforce

col1 head	col2 head	col3 head
col1 text	col2 text	col3 text
col1 text	col2 text	col3 text
col1 text	col2 text	col3 text

Section title of second appendix

Fusce mauris. Vestibulum luctus nibh at lectus. Sed bibendum, nulla a faucibus semper, leo velit ultricies tellus, ac venenatis arcu wisi vel nisl. Vestibulum diam. Aliquam pellentesque, augue quis sagittis posuere, turpis lacus congue quam, in hendrerit risus eros eget felis. Maecenas eget erat in sapien mattis porttitor. Vestibulum porttitor. Nulla facilisi. Sed a turpis eu lacus commodo facilisis. Morbi fringilla, wisi in dignissim interdum, justo lectus sagittis dui, et vehicula libero dui cursus dui. Mauris tempor ligula sed lacus. Duis cursus enim ut augue. Cras ac magna. Cras nulla.

Subsection title of second appendix

Sed commodo posuere pede. Mauris ut est. Ut quis purus. Sed ac odio. Sed vehicula hendrerit sem. Duis non odio. Morbi ut dui. Sed accumsan risus eget odio. In hac habitasse platea dictumst. Pellentesque non elit. Fusce sed justo eu urna porta tincidunt. Mauris felis odio, sollicitudin sed, volutpat a, ornare ac, erat. Morbi quis dolor. Donec pellentesque, erat ac sagittis semper, nunc dui lobortis purus, quis congue purus metus ultricies tellus. Proin et quam. Class aptent taciti sociosqu ad litora torquent per conubia nostra, per inceptos hymenaeos. Praesent sapien turpis, fermentum vel, eleifend faucibus, vehicula eu, lacus.

Sed commodo posuere pede. Mauris ut est. Ut quis purus. Sed ac odio. Sed vehicula hendrerit sem. Duis non odio. Morbi ut dui. Sed accumsan risus eget odio. In hac habitasse platea dictumst. Pellentesque non elit. Fusce sed justo eu urna porta tincidunt. Mauris felis odio, sollicitudin sed, volutpat a, ornare ac, erat. Morbi quis dolor. Donec pellentesque, erat ac sagittis semper, nunc dui lobortis purus, quis congue purus metus ultricies tellus. Proin et quam. Class aptent taciti sociosqu ad litora torquent per conubia nostra, per inceptos hymenaeos. Praesent sapien turpis, fermentum vel, eleifend faucibus, vehicula eu, lacus.

Fig. 4. This is an example for appendix figure

Subsubsection title of second appendix

Lorem ipsum dolor sit amet, consectetuer adipiscing elit. Ut purus elit, vestibulum ut, placerat ac, adipiscing vitae, felis. Curabitur dictum gravida mauris. Nam arcu libero, nonummy eget, consectetuer id, vulputate a, magna. Donec vehicula augue eu neque.

Example for an equation inside the appendix:

$$p = \frac{\gamma^2 - (n_C - 1)H}{(n_C - 1) + H - 2\gamma}, \tag{4}$$

$$\theta = \frac{(\gamma - H)^2(\gamma - n_C - 1)^2}{(n_C - 1 + H - 2\gamma)^2}. \tag{5}$$

Example of another appendix section

Nam dui ligula, fringilla a, euismod sodales, sollicitudin vel, wisi. Morbi auctor lorem non justo. Nam lacus libero, pretium at, lobortis vitae, ultricies et, tellus. Donec aliquet, tortor sed accumsan bibendum, erat ligula aliquet magna, vitae ornare odio metus a mi. Morbi ac orci et nisl hendrerit mollis. Suspendisse ut massa. Cras nec ante. Pellentesque a nulla. Cum sociis natoque penatibus et magnis dis parturient montes, nascetur ridiculus mus. Aliquam tincidunt urna. Nulla ullamcorper vestibulum turpis. Pellentesque cursus luctus mauris

$$\mathcal{L} = i\bar{\psi}\gamma^\mu D_\mu \psi - \frac{1}{4} F_{\mu\nu}^a F^{a\mu\nu} - m\bar{\psi}\psi. \tag{6}$$

Nulla malesuada porttitor diam. Donec felis erat, congue non, volutpat at, tincidunt tristique, libero. Vivamus viverra fermentum felis. Donec nonummy pellentesque ante. Phasellus adipiscing semper elit. Proin fermentum massa ac quam. Sed diam turpis, molestie vitae, placerat a, molestie nec, leo. Maecenas lacinia. Nam ipsum ligula, eleifend at, accumsan nec, suscipit a, ipsum. Morbi blandit ligula feugiat magna. Nunc eleifend consequat lorem. Sed lacinia nulla vitae enim. Pellentesque tincidunt purus vel magna. Integer non enim. Praesent euismod nunc eu purus. Donec bibendum quam in tellus. Nullam cursus pulvinar lectus. Donec et mi. Nam vulputate metus eu enim. Vestibulum pellentesque felis eu massa.

Nulla malesuada porttitor diam. Donec felis erat, congue non, volutpat at, tincidunt tristique, libero. Vivamus viverra fermentum felis. Donec nonummy pellentesque ante. Phasellus adipiscing semper elit. Proin fermentum massa ac quam. Sed diam turpis, molestie vitae, placerat a, molestie nec, leo. Maecenas lacinia. Nam ipsum ligula, eleifend at, accumsan nec, suscipit a, ipsum. Morbi blandit ligula feugiat magna. Nunc eleifend consequat lorem. Sed lacinia nulla vitae enim. Pellentesque tincidunt purus vel magna. Integer non enim. Praesent euismod nunc eu purus. Donec bibendum quam in tellus. Nullam cursus pulvinar lectus. Donec et mi. Nam vulputate metus eu enim. Vestibulum pellentesque felis eu massa.

Nulla malesuada porttitor diam. Donec felis erat, congue non, volutpat at, tincidunt tristique, libero. Vivamus viverra fermentum felis. Donec nonummy pellentesque ante. Phasellus adipiscing semper elit. Proin fermentum massa ac quam. Sed diam turpis, molestie vitae, placerat a, molestie nec, leo. Maecenas lacinia. Nam ipsum ligula, eleifend at, accumsan nec, suscipit a, ipsum. Morbi blandit ligula feugiat magna. Nunc eleifend consequat lorem. Sed lacinia nulla vitae enim. Pellentesque tincidunt purus vel magna. Integer non enim. Praesent euismod nunc eu purus. Donec bibendum quam in tellus. Nullam cursus pulvinar lectus. Donec et mi. Nam vulputate metus eu enim. Vestibulum pellentesque felis eu massa.

Table 5.

col1 head	col2 head	col3 head
col1 text	col2 text	col3 text
col1 text	col2 text	col3 text
col1 text	col2 text	col3 text

Nulla malesuada porttitor diam. Donec felis erat, congue non, volutpat at, tincidunt tristique, libero. Vivamus viverra fermentum felis. Donec nonummy pellentesque ante. Phasellus adipiscing semper elit. Proin fermentum massa ac quam. Sed diam turpis, molestie vitae, placerat a, molestie nec, leo. Maecenas lacinia. Nam ipsum ligula, eleifend at, accumsan nec, suscipit a, ipsum. Morbi blandit ligula feugiat magna. Nunc eleifend consequat lorem. Sed lacinia nulla vitae enim. Pellentesque tincidunt purus vel magna. Integer non enim. Praesent euismod nunc eu purus. Donec bibendum quam in tellus. Nullam cursus pulvinar lectus. Donec et mi. Nam vulputate metus eu enim. Vestibulum pellentesque felis eu massa. Donec bibendum quam in tellus. Nullam cursus pulvinar lectus. Donec et mi. Nam vulputate metus eu enim. Vestibulum pellentesque felis eu massa.

Competing interests

No competing interest is declared.

Author contributions statement

Must include all authors, identified by initials, for example: S.R. and D.A. conceived the experiment(s), S.R. conducted the experiment(s), S.R. and D.A. analysed the results. S.R. and D.A. wrote and reviewed the manuscript.

Acknowledgments

The authors thank the anonymous reviewers for their valuable suggestions. This work is supported in part by funds from the National Science Foundation (NSF: # 1636933 and # 1920920).

References

D. Bahdanau, K. Cho, and Y. Bengio. Neural machine translation by jointly learning to align and translate. *arXiv preprint arXiv:1409.0473*, 2014.

S. Horvath and K. Raj. Dna methylation-based biomarkers and the epigenetic clock theory of ageing. *Nature Reviews Genetics*, 19(6):371, 2018.

M. T. Imboden, M. P. Harber, M. H. Whaley, W. H. Finch, D. L. Bishop, and L. A. Kaminsky. Cardiorespiratory fitness and mortality in healthy men and women. *Journal of the American College of Cardiology*, 72(19):2283–2292, 2018.

S. Ji, W. Xu, M. Yang, and K. Yu. 3d convolutional neural networks for human action recognition. *IEEE Transactions on Pattern Analysis and Machine Intelligence*, 35(1):221–231, 2012.

A. Krizhevsky, I. Sutskever, and G. E. Hinton. Imagenet classification with deep convolutional neural networks. In *Advances in Neural Information Processing Systems*, pages 1097–1105, 2012.

Y. LeCun, Y. Bengio, and G. Hinton. Deep learning. *Nature*, 521(7553):436, 2015.

S. Motiian, M. Piccirilli, D. A. Adjeroh, and G. Doretto. Unified deep supervised domain adaptation and generalization. In *Proceedings of the IEEE International Conference on Computer Vision*, pages 5715–5725, 2017.

K. P. Murphy. *Machine learning: A probabilistic perspective.* MIT press, 2012.

T. V. Pyrkov, E. Getmantsev, B. Zhurov, K. Avchaciov, M. Pyatnitskiy, L. Menshikov, K. Khodova, A. V. Gudkov, and P. O. Fedichev. Quantitative characterization of biological age and frailty based on locomotor activity records. *Aging (Albany NY)*, 10(10):2973, 2018.

S. A. Rahman and D. Abdul. Centroid of age neighborhoods: A generalized approach to estimate biological age. In *2019 IEEE EMBS International Conference on Biomedical & Health Informatics (BHI)*, pages 1–4. IEEE, 2019.

D. Ravì, C. Wong, F. Deligianni, M. Berthelot, J. Andreu-Perez, B. Lo, and G.-Z. Yang. Deep learning for health informatics. *IEEE Journal of Biomedical and Health Informatics*, 21(1):4–21, 2016.

Z. Wang, X. Tang, W. Luo, and S. Gao. Face aging with identity-preserved conditional generative adversarial networks. In *Proceedings of the IEEE Conference on Computer Vision and Pattern Recognition*, pages 7939–7947, 2018.

K. Zhang, N. Liu, X. Yuan, X. Guo, C. Gao, and Z. Zhao. Fine-grained age estimation in the wild with attention LSTM networks. *arXiv preprint arXiv:1805.10445*, 2018.

Author Name. This is sample author biography text. The values provided in the optional argument are meant for sample purposes. There is no need to include the width and height of an image in the optional argument for live articles. This is sample author biography text this is sample author biography text this is sample author biography text this is sample author biography text this is sample author biography text this is sample author biography text this is sample author biography text this is sample author biography text.

Author Name. This is sample author biography text this is sample author biography text this is sample author biography text this is sample author biography text this is sample author biography text this is sample author biography text this is sample author biography text this is sample author biography text.
